# Matrix metalloproteinases (MMPs) in fresh human prostate tumour tissue and organ-cultured prostate tissue: Levels of collagenolytic and gelatinolytic MMPs are low, variable and different in fresh tissue versus organ-cultured tissue

**DOI:** 10.1054/bjoc.2000.1712

**Published:** 2001-04

**Authors:** J Varani, Y Hattori, M K Dame, T Schmidt, H S Murphy, K J Johnson, K J Wojno

**Affiliations:** The Department of Pathology, The University of Michigan Medical School, Ann Arbor, Michigan, 48109; Department of Pathology and Laboratory Medicine, VAMC – Ann Arbor, Ann Arbor, Michigan, 48105; The Department of Pathology, St. John Hospital and Medical Center, Detroit, Michigan, 48236-2172

**Keywords:** prostate, carcinoma, organ culture, matrix metalloproteinases, collagenase, gelatinase

## Abstract

Prostate tissue was obtained from 22 radical prostatectomies (performed for clinical management of prostate carcinoma) immediately after surgery. A small piece of tissue was fixed immediately in formalin and used for routine histology while a second piece was frozen in OCT and used for immuno-histochemistry. Another small piece was used for isolation of epithelial and stromal cells. The remainder of the tissue was cut into 2 × 2 mm pieces and incubated in organ culture for 8 days. In organ culture, non-malignant, basal epithelial cells underwent a proliferative response. This was accompanied by de-differentiation of glandular structures and by migration of epithelial cells across the surface of the tissue. Erosion of the basement membrane could also be seen in places, but was not widespread. Invasion of epithelial cells into the adjacent stroma was not evident. Production of matrix metalloproteinases (MMPs) with gelatinolytic activity or collagenolytic activity was assessed in organ culture and compared to expression patterns in fresh tissue. MMP-1 (interstitial collagenase) and MMP-9 (92-kDa gelatinase B) were undetectable or low in fresh tissue specimens. Both enzymes were detected in organ culture and both increased over time. Even after 6 days, however, there was only a low level of gelatin-hydrolytic activity and no measurable collagen-hydrolytic activity. In past studies we used organ cultures of normal skin and malignant skin tumours (basal cell carcinomas) to help elucidate the role of collagenolytic and gelatinolytic MMPs in epithelial cell invasion (Varani et al, 2000). Compared to MMP levels observed in skin, levels of these enzymes in prostate are low. The low level of collagenolytic and gelatinolytic MMPs in fresh prostate tissue and in organ-cultured prostate tissue may help explain why there is little tissue destruction in many primary prostate tumours and why the majority of such tumours remain confined to the prostate for extended periods. © 2001 Cancer Research Campaign http://www.bjcancer.com


*British Journal of Cancer* (2001) **84**(8), 1076–1083
© 2001 Cancer Research Campaign
doi: 10.1054/ bjoc.2000.1712, available online at http://www.idealibrary.com on IDEAL®

http://www.bjcancer.com

### Matrix metalloproteinases (MMPs) in fresh human prostate tumour tissue and organ-cultured prostate tissue: Levels of collagenolytic and gelatinolytic MMPs are low, variable and different in fresh tissue versus organ-cultured tissue

**J Varani[1], Y Hattori[1], MK Dame[1], T Schmidt[1], HS Murphy[2], KJ Johnson[1] and KJ Wojno[3]**

[1]The Department of Pathology, The University of Michigan Medical School, Ann Arbor, Michigan 48109; [2]Department of Pathology and Laboratory Medicine, VAMC – Ann Arbor, Ann Arbor, Michigan 48105; [3]The Department of Pathology, St. John Hospital and Medical Center, Detroit, Michigan 48236-2172

**Summary** Prostate tissue was obtained from 22 radical prostatectomies (performed for clinical management of prostate carcinoma) immediately after surgery. A small piece of tissue was fixed immediately in formalin and used for routine histology while a second piece was frozen in OCT and used for immuno-histochemistry. Another small piece was used for isolation of epithelial and stromal cells. The remainder of the tissue was cut into 2 × 2 mm pieces and incubated in organ culture for 8 days. In organ culture, non-malignant, basal epithelial cells underwent a proliferative response. This was accompanied by de-differentiation of glandular structures and by migration of epithelial cells across the surface of the tissue. Erosion of the basement membrane could also be seen in places, but was not widespread. Invasion of epithelial cells into the adjacent stroma was not evident. Production of matrix metalloproteinases (MMPs) with gelatinolytic activity or collagenolytic activity was assessed in organ culture and compared to expression patterns in fresh tissue. MMP-1 (interstitial collagenase) and MMP-9 (92-kDa gelatinase B) were undetectable or low in fresh tissue specimens. Both enzymes were detected in organ culture and both increased over time. Even after 6 days, however, there was only a low level of gelatin-hydrolytic activity and no measurable collagen-hydrolytic activity. In past studies we used organ cultures of normal skin and malignant skin tumours (basal cell carcinomas) to help elucidate the role of collagenolytic and gelatinolytic MMPs in epithelial cell invasion (Varani et al, 2000). Compared to MMP levels observed in skin, levels of these enzymes in prostate are low. The low level of collagenolytic and gelatinolytic MMPs in fresh prostate tissue and in organ-cultured prostate tissue may help explain why there is little tissue destruction in many primary prostate tumours and why the majority of such tumours remain confined to the prostate for extended periods. © 2001 Cancer Research Campaign http://www.bjcancer.com

**Keywords**: prostate; carcinoma; organ culture; matrix metalloproteinases; collagenase; gelatinase

The matrix metalloproteinases (MMPs) are a family of genetically related hydrolytic enzymes that together have the capacity to degrade virtually every component of the extracellular matrix (Khokha and Denhardt, 1989; Murphy and Docherty, 1992; Crawford and Matrisian, 1996). A role for MMPs in experimental tumour invasion and metastasis is suggested by a variety of evidence (Khokha et al, 1989; DeClerck et al, 1991; Alexander and Werb, 1992; DeClerck et al, 1992; Cha et al, 1996; Imren et al, 1996). Although there is little direct evidence for MMP function in human tumour spread under in situ conditions, MMP expression is elevated in various human tumours relative to the normal counterpart tissues (McDonnell et al, 1991; Muller et al, 1991, 1993; Gray et al, 1992; Pyke et al, 1992; Assam et al, 1993; Majmudar et al, 1994; MacDougall et al, 1995; Murray et al, 1996; Johansson et al, 1997).

In regards to prostate cancer specifically, Hamdy et al (1994) and Festuccia et al (1996) reported elevated levels of MMP-9 (92-kDa gelatinase B) in prostate tumour tissue relative to normal prostate or benign prostate hyperplasia (BPH). Not all investigators have demonstrated this relationship, however. Lokeshwar et al (1993) found higher MMP-9 expression in BPH than in prostate cancer, but higher MMP-2 expression in tumour tissue. Stearns and Wang (1993), Montironi et al (1995, 1996), and Still et al (2000) also demonstrated increased MMP-2 (72-kD gelatinase A) expression in prostate carcinoma, while Upadhyay et al (1999) found no overall change in level of MMP-2 expression between normal and malignant prostate tissue but a change in distribution pattern. In contrast to the expression of these gelatinolytic enzymes (with type IV collagenolytic activity), there is little evidence for elaboration of enzymes which can degrade interstitial connective tissue. Pajouh et al (1991) reported that mRNA for MMP-1 (interstitial collagenase) was not detectable in either normal or malignant prostate specimens. MMP-1 immunoreactivity was detected in plasma of prostate cancer patients, but the amount detected was not significantly different from amount present in normal volunteers (Jung et al, 1997). Finally, Wilson et al (1993) identified enzymes with caseinolytic activity (consistent with the action of mammalian collagenases) in prostatic secretions of individuals with BPH, atypia or prostate cancer. Considerable variability was seen, and there was no clear-cut relationship with disease-state.

*Received 21 August 2000*
*Revised 14 December 2000*
*Accepted 19 December 2000*

*Correspondence to:* J Varani

Although these past studies clearly indicate that several MMPs are elaborated in prostate tumours and suggest differences between malignant and non-malignant tissue, the heterogeneity in prostate tumour presentation and the variability in the analytical methods used make elucidating the relationship between enzyme elaboration and disease-state difficult to ascertain. Furthermore, the presence of active forms of the enzymes has not been established in these studies, and the biological significance of the reported differences is unclear. In the present study, a combination of approaches has been employed to assess expression of collagenolytic and gelatinolytic MMPs in prostate tissue removed for treatment of prostate carcinoma. We report here that gelatinolytic and collagenolytic MMPs can be detected in freshly isolated prostate tissue or in short-term (2–3 day) organ culture. Expression is heterogeneous from tissue to tissue, consistent with the variation in disease presentation. Expression patterns in fresh tissue and organ-cultured tissue also differ. Enzyme levels measured in vitro appear to primarily reflect production by non-malignant components rather than malignant cells. Perhaps most interesting, overall levels of the collagenolytic and gelatinoltyic MMPs are low in prostate tissue relative to other tumours (for example, basal cell carcinomas of skin) that have been examined in the past with the same approaches (Varani et al, 2000). The lack of tissue destruction and the relatively low invasive potential of the majority of prostate tumours may be reflective of this.

### MATERIALS AND METHODS

### Tissue source

Human prostate tissue was obtained from 22 radical prostatectomies immediately after surgery. In all cases, a diagnosis of prostate carcinoma had been previously made based on a 6 needle biopsy protocol, and in all cases there was no evidence of tumour spread beyond the prostate at the time of surgery. 5 of the tumours were classified as Gleason grades 4 or 5; 13 were classified as Gleason grade 6 or 7; and the remaining 4 were of higher grades (grades 8–10). The percentage of the tumour component within the tissue ranged from 1–90%. Routinely, we were able to obtain 1–2 $cm^3$ of tissue from areas of the prostate in which tumour was present. In addition, areas of the prostate in which no tumour was identified histologically were also available in most cases. Prostate intra-epithelial neoplasm (PIN) and/or BPH were present in some of these tissue specimens. Upon arrival in the laboratory, a small piece from each specimen was fixed in 10% buffered formalin, stained with haematoxylin and eosin and evaluated for amount of tumour present and histological features of the tumour. A second tissue piece was frozen in OCT immediately after surgery and used for immunostaining. Additional tissue pieces were used for the isolation of epithelial cells and stromal (fibroblast-like) cells in monolayer culture (see below) and the remainder was incubated in organ culture (see below).

### MMP immunohistology

Tissue frozen in OCT was examined by immunoperoxidase staining for MMP-1, MMP-2, MMP-9 and MMP-13 expression as described previously (Varani et al, 2000). Briefly, frozen sections were mounted on glass slides coated with poly-L-lysine and stained using the ABC method (Vector Labs; Burlingame, CA). Diaminobenzidine was used as the chromogenic substrate, and tissue were counterstained with haematoxylin. A rabbit polyclonal IgG antibody to MMP-1 was obtained from Chemicon (Temecula, CA). Monoclonal antibodies to MMP-2, MMP-9 and MMP-13 were obtained from Oncogene Sciences (Cambridge, MA). Antibody specificity was established based on reactivity with purified MMPs in Western blots, reactivity with appropriately sized moieties in organ culture fluids and a lack of reactivity with other sized moieties in the same culture fluids (Varani et al, 2000). A mouse monoclonal IgG1 antibody (Oncogene Sciences) and a rabbit polyclonal IgG antibody (R & D Systems, Minneapolis, MN) were used as controls.

### Organ culture procedures and assessment

Tissue for organ culture was cut into pieces approximately 1.5–2 mm in size and incubated in wells of a 24-well dish; each well contained 5–6 tissue pieces in 0.5 ml of culture medium. The culture medium was Keratinocyte Growth Medium (KGM) (Clonetics, Inc, Walkersville, MD). KGM is a serum-free modification of MCDB-153 medium, supplemented with a combination of growth-promoting agents including epidermal growth factor (0.1 ng $ml^{-1}$), insulin (0.5 µg $ml^{-1}$) and pituitary extract (2%). It was further supplemented with $CaCl_2$ to bring the final $Ca^{2+}$ concentration to 1.4 mM and with 800 ng $ml^{-1}$ dihydrotestosterone (DHT). Cultures were incubated at 37˚C in an atmosphere containing 5% $CO_2$ and 95% air. Fresh culture medium was provided at 2–3 day intervals.

At the end of the incubation period (day 8), tissue pieces were fixed in 10% buffered formalin and stained with haematoxylin and eosin. Histological features were compared to those seen at time-zero. Sections from the same tissue pieces were also stained with an anti-cytokeratin antibody (K903), from Boehringer Mannheim Biochemicals (Indianapolis IN). This antibody recognizes high molecular weight keratins expressed by normal basal epithelial cells in the prostate but not expressed in prostate carcinoma cells (Makin et al, 1984). Additional sections were stained with an antibody to the proliferation-associated antigen, Ki-67 (Key et al, 1993). This antibody (MIB-1) was obtained from Immunotech (Westbrook, ME). Ki-67 staining was used to confirm that epithelial cell expansion did, in fact, reflect a proliferative response. Finally, sections were stained by the Periodic Acid-Schiff (PAS) method and used for basement membrane evaluation. The percentage of the gland surrounded by PAS-positive material was estimated and scored as 0, 0.25, 0.5, 0.75 or 1.0, where 0 indicates a complete lack of PAS-positive material around the gland and 1.0 indicates a complete encompassing of the gland with PAS-reactive material. Some sections were also stained with PAS-methenamine silver to confirm PAS results. PAS-methenamine silver is more specific for basement membrane, although cellular architecture is lost (Varani et al, 1999).

Used organ culture fluid was collected at each media change and analysed for MMP-1, MMP-2, MMP-9 and MMP-13 by a combination of zymography, Western blotting and specific substrate degradation assays (see below).

### Isolation of cells from prostate tissue

Prostate epithelial cells and stromal cells were isolated and characterized as described in a recent report (Varani et al, 1999). Epithelial cells were defined on the basis of cobblestone morphology. Normal

© 2001 Cancer Research Campaign basal epithelial cells and malignant epithelial cells were distinguished on the basis of reactivity with anti-keratin antibody K903 (Makin et al, 1984). Virtually all of the epithelial cells that grew in monolayer culture were strongly positive (indicative of normal basal cells). Stromal cells were identified on the basis of spindle-shape and a lack of staining with keratin antibodies. Of the stromal cells isolated, approximately 5–10% were identified as smooth muscle cells on the basis of reactivity with desmin and smooth muscle α-actin and 10–15% were identified as myofibroblasts on the basis of reactivity with smooth muscle α-actin but not desmin. Occasional cells (<1%) stained with antibody to von Willibrand factor and were identified as endothelial cells. The remaining cells stained with none of these markers and were identified as fibroblasts.

### Substrate-embedded enzymography

SDS-PAGE substrate-embedded enzymography (zymography) was used to identify enzymes with collagenase and gelatinase activities. Assays were carried out exactly as described in a previous report (Gibbs et al, 1999). Briefly, denatured but non-reduced culture fluid samples were resolved in 7.5% SDS-PAGE gels prepared with the added incorporation of gelatin (1 mg $ml^{-1}$) or β-casein (1 mg $ml^{-1}$) prior to casting. After electrophoresis, gels were washed twice for 15 min in 50 mM Tris buffer containing 1 mM $Ca^{2+}$ and 0.5 mM $Zn^{2+}$ with 2.5% Triton X-100. The gels were then incubated overnight in Tris buffer with 1% Triton X-100 and stained the following day with Coumassie Brilliant Blue 250-R. Following destaining, zones of enzyme activity were detected as regions of negative staining against the dark background. Volumes of 5–35 μl of undiluted specimen were normally used for these assays; zones of activity were proportional to the quantity of culture fluid used.

### Western blotting

Culture fluid proteins were resolved by SDS-PAGE and transferred to nitrocellulose filters. Following treatment of filters with specific antibodies, the filters were reacted with horseradish peroxidase (HRP)-conjugated secondary antibodies. Reactive proteins were detected by enhanced chemiluminescence (ECL, Amersham) after treatment with luminol and visualization on light-sensitive autoradiography film (Fisher et al, 1996).

### Gelatin and collagen degradation assays

Acid-solubilized (rat tail) fibrillar type I collagen was obtained from Becton-Dickinson (Bedford, MA). Native collagen and heat-denatured (60˚C 5 min) collagen (i.e., gelatin) were diluted to 1 mg $ml^{-1}$ in phosphate-buffered saline (PBS, pH 7.2), and incubated at 37˚C for 18 hours with prostate organ culture fluids which had previously been concentrated 10X. Prior to the addition of the collagen or gelatin substrates, culture fluids were exposed to 1 mM aminophenyl mercuric acetate (APMA) for 90 min to activate latent enzyme (Gibbs et al, 1999). Substrates not exposed to organ culture fluids and culture fluids without substrates were incubated in parallel as negative controls. At the end of the 18-hour incubation period, samples were resolved by SDS-PAGE. The presence of active enzyme was determined, based on the disappearance of the parental α1(I) and α2(I) collagen (or gelatin) bands and the appearance of lower molecular weight fragments (Mulligan et al, 1993; Varani et al, 2000).

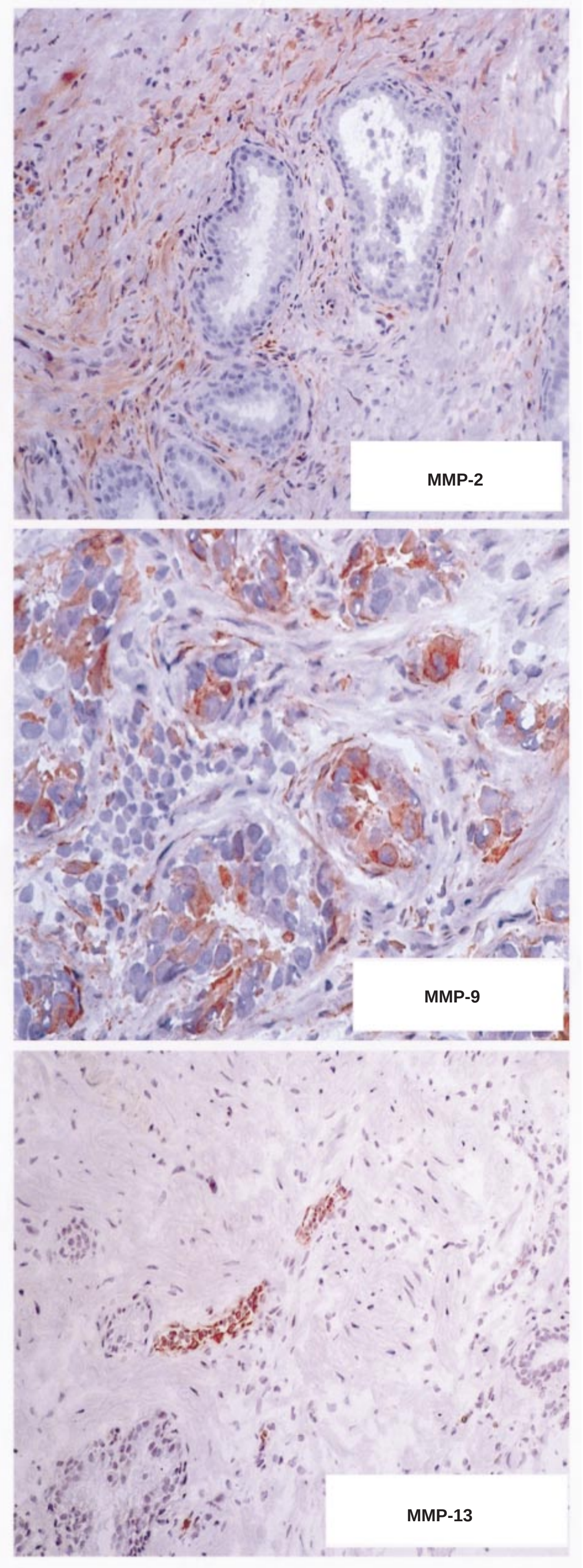

**Figure 1** MMP expression in prostate tissue immediately following surgery. Tissue frozen in OCT was incubated with monoclonal antibodies to each of the 3 MMPs followed by incubation with an immunoperoxidase-conjugated secondary antibody. Diaminobenzidine was used as chromogen and haematoxylin was used as counterstain. (Magnification: MMP-2, ×160; MMP-9, ×400; MMP-13; ×160)

© 2001 Cancer Research Campaign

### RESULTS

### MMP expression in human prostate tumour tissue immediately after surgery

Immunoperoxidase staining was used to determine expression of enzymes with gelatinolytic activity (i.e., MMP-2 and MMP-9) in 22 different human prostate tumour specimens immediately after surgery. In 9 of the specimens, regardless of whether or not tumour was present or the amount present, there was little or no detectable staining for MMP-2. MMP-2 reactivity was observed in the remainder of the specimens. MMP-2 reactivity was visible throughout the stroma in the majority of these specimens (Figure 1, upper panel). In some specimens, reactivity was also observed in a small number of normal or abnormal glandular epithelial cells. Routinely, however, epithelial cells were negative for MMP-2 reactivity.

MMP-9 immunoreactivity was also weak or undetectable in many of the specimens (11 of 22). In other specimens, staining with anti-MMP-9 revealed the presence of antigen in either or both stroma and epithelial structures. The most intense staining with anti-MMP-9 occurred in the epithelial cells of highly anaplastic lesions, where glandular structures were disorganized (Figure 1, middle panel).

Tissue sections from the same specimens were also stained with antibodies to MMP-1 (interstitial collagenase) and MMP-13 (collagenase 3). With the exception of an occasionally positive stromal cell, no staining with antibody to MMP-1 was observed in any of the specimens (not shown). In contrast, staining with anti-MMP-13 was seen in virtually every specimen. Staining was confined to vascular structures as shown in the lower panel of Figure 1. Overall, there was no clear distinction in MMP-13 immunoreactivity between tissue sections in which extensive tumour was present and sections in which there was little or no evidence of tumour.

### Human prostate tissue in organ culture: histological features

Tissue pieces from each surgical specimen were incubated in organ culture as described in the Materials and Methods section. After 8 days, the tissue was fixed in 10% buffered formalin and evaluated histologically. Consistent with what we have reported previously (Varani et al, 1999), there was a loss of secretory epithelial cells and replacement of the normal glandular structure with morphologically undifferentiated (basal) epithelial cells. In some places, basal epithelial cells filled the lumens of the glands, obliterating normal architectural features. In addition to occupying the glands, epithelial cells also grew out from the glands to cover the surface of the tissue. Figure 2A shows the typical appearance of haematoxylin and eosin-stained tissue on day 8. Staining with antibody to Ki-67 confirmed a proliferative response in the epithelial cell population (Figure 2B). Staining with the anti-keratin antibody, K903, revealed strong reactivity in virtually all of the hyper-proliferative glands, as well as in the cells which migrated out from the glands (Figure 2C). Reactivity of the proliferating cells with this antibody, which is known to react with basal epithelial cells but not with malignant cells (Makin et al, 1984), indicates that the proliferating cells were derived from the non-malignant epithelial cell component present in the original tissue. Consistent with what we have reported in the past (Varani et al, 1999), the vast majority of histologically abnormal, K903-negative cells (presumably tumour) were lost from the tissue after 8 days in organ culture under the conditions employed here.

Figure 2D and 2E show the appearance of the tissue after staining by the PAS method. Consistent with the picture shown in

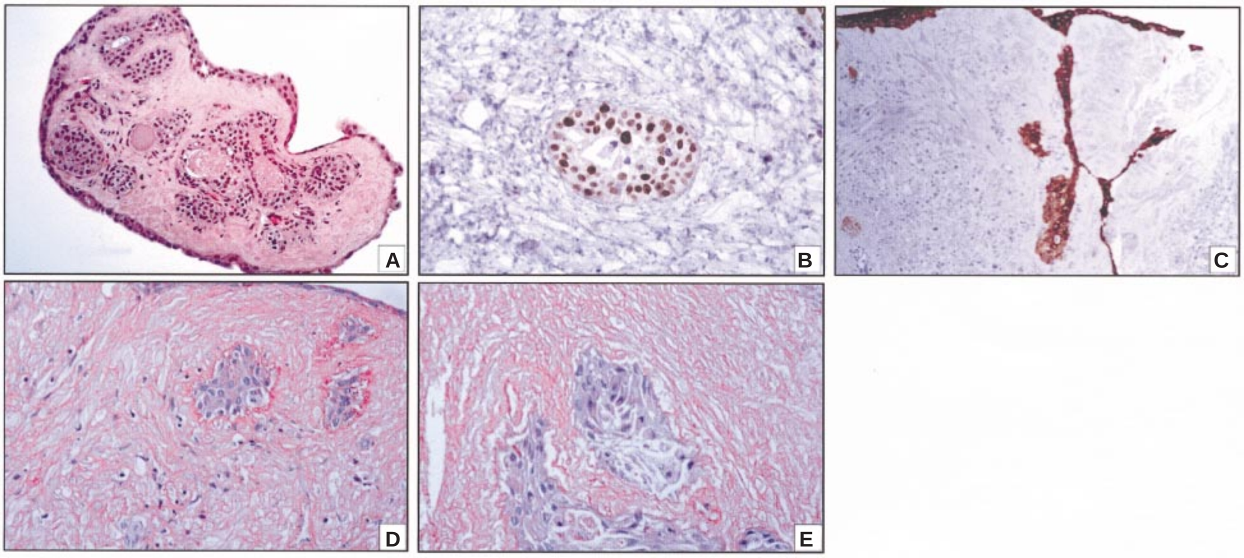

**Figure 2** Histological appearance of human prostate tissue after 8 days in organ culture. (**A**) Haematoxylin and eosin-stained section showing glandular structures in which secretory cells have disappeared and basal epithelial cells have filled in the lumens of the glands. Epithelial cells also cover the surface of the tissue piece. (**B**) Ki-67-stained section demonstrating proliferation in many of the epithelial cells. (**C**) K903-stained section showing strong reactivity in the hyper-proliferative glands and in cells that have migrated out of these glands. (**D**) PAS-stained section showing an epithelial structure surrounded by distinct PAS-positive (basement membrane) material. (**E**) PAS-stained section showing epithelial cells not surrounded by distinct PAS-positive (basement membrane) material

© 2001 Cancer Research Campaign

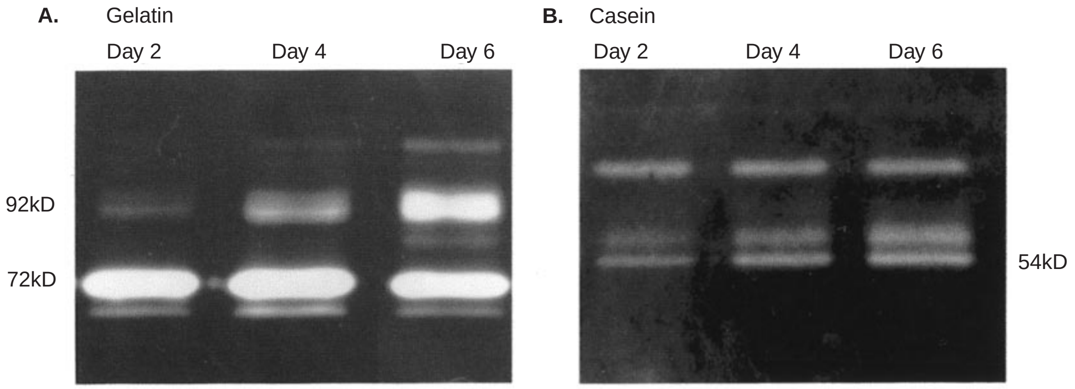

**Figure 3** Gelatinolytic and β- caseinolytic activity assessed by zymography in 48-hour prostate organ culture fluids obtained after 2, 4 or 6 days in culture. (**A**) Gelatin: Pro-MMP-2 (latent form) is represented by the major band at 72 kDa. The shadow band immediately beneath the major band represents a processed (active) form of the enzyme. MMP-2 remains consistent over the 6-day period. Pro-MMP-9 (latent form) is represented by the major band at 92 kDa. The shadow band immediately beneath the major band represents a processed (active) form of the enzyme. MMP-9 increases over the 6-day period. (**B**) β-casein: Pro-MMP-1 is represented by a doublet in the 54–57 kDa region of the gel. There are no lower molecular weight forms attributable to the active form of the enzyme. MMP-1 increases over the 6-day period

Figure 2D, most of the hyper-proliferative glands were surrounded by PAS-reactive material (basement membrane). However, in certain of the glands (Figure 2E), there were areas in which PAS-reactivity was not evident. Based on a semi-quantitative assessment of 77 separate glands from 9 different tissue specimens (after 8 days in organ culture), the fraction of the glandular periphery that was clearly delineated from the surrounding stroma by PAS-positive material was 0.8 ± 0.15% (compared to approximately 0.9 ± 0.10 in fresh tissue; not statistically different at $P < 0.05$ level). Selected specimens were also stained with PAS-methenamine silver, which is a more specific basement membrane stain (Varani et al, 1999). PAS-staining and staining with PAS-methenamine silver occurred in parallel (not shown).

### MMP expression in human prostate tissue in organ culture

Prostate tissue was incubated in organ culture for 8 days. Culture fluids were collected on days 2, 4 and 6, and assessed for MMP-2 and MMP-9 by gelatin zymography. Zones of activity corresponding to both enzymes were seen at all 3 time points. The level of MMP-2 remained constant throughout the 6-day culture period (Figure 3A). In contrast, the level of MMP-9 was low initially, but increased as a function of time in culture (Figure 3A). With both enzymes, the higher molecular weight, latent, proenzyme forms predominated. Lower molecular weight forms, characteristic of active enzymes, were detectable in most cases, but these forms were present as thin 'shadow' bands immediately below the parent enzyme bands, and constituted a small fraction of the total.

The same culture fluids were analysed for β-caseinolytic activity. Activity was detected as a doublet in the 54 kD region of the gel (indicative of the proform of MMP-1). Essentially no activity was seen in the 45 kD region of the gel (corresponding to active form of the enzyme). As can be seen in Figure 3B, there was little activity in day 2 organ culture fluids, with progressively increased amounts on day 4 and day 6. The β-caseinolytic activity in the prostate organ culture fluids was due mainly to MMP-1 based on the following information: i) activity was completely abolished by overnight incubation of the zymogram in buffer containing 10 mM EDTA (metalloproteinase inhibitor) but was unaffected by the presence of 2 mM phenyl methyl sulphonylfluoride (serine proteinase inhibitor) in the buffer; ii) upon exposure of the culture fluid to APMA, an agent used routinely as an MMP activator (Gibbs et al, 1999), a 'band-shift' to 45 kD occurred; iii) β-caseinolytic activity in the prostate organ culture fluid co-migrated with a zone of β-caseinolytic activity produced by human dermal fibroblast MMP-1; and iv) Western blotting revealed the presence of an anti-MMP-1-reactive moiety in prostate organ culture fluids that co-migrated with the β-caseinolytic activity (data not shown). In contrast, no reactivity with an antibody to MMP-13 was detected by Western blotting in the same specimens. In addition to the β-caseinolytic activity observed in the 54 kD region of the gel, a thin band of activity was also detected in the higher molecular weight region (Figure 3B). This activity co-migrated with human dermal fibroblast MMP-2. No other zones of activity were detected.

Organ culture fluids were obtained from prostate tissues after incubation for 72 hours and concentrated 10-fold. Gelatin and collagen degradation assays were carried out using the concentrated culture fluid. A low level of gelatin fragmentation was detected in these culture fluids, while fragmentation of intact fibrillar collagen was not evident with either non-activated culture fluids or following exposure to 1 mM APMA (Figure 4). Organ culture fluids collected on day 6 (72-hour culture fluids) and handled the same way also were devoid of detectable collagenolytic activity (not shown).

In a final set of studies, epithelial cells and stromal cells were grown for 2 days in monolayer culture. Cell culture fluids obtained at the end of the incubation period were examined by gelatin and β-casein zymography. Epithelial cells produced gelatinolytic activities corresponding to both MMP-2 and MMP-9 while stromal fibroblast-like cells elaborated only MMP-2. Both cell types also produced a 54 kD β-caseinolytic activity. The level of β-caseinolytic activity was higher in the culture fluid from epithelial cells than in the stromal cell culture fluid. There was no evidence of a higher molecular weight activity that would be consistent with MMP-13 in the culture fluid from either cell type (not shown).

© 2001 Cancer Research Campaign

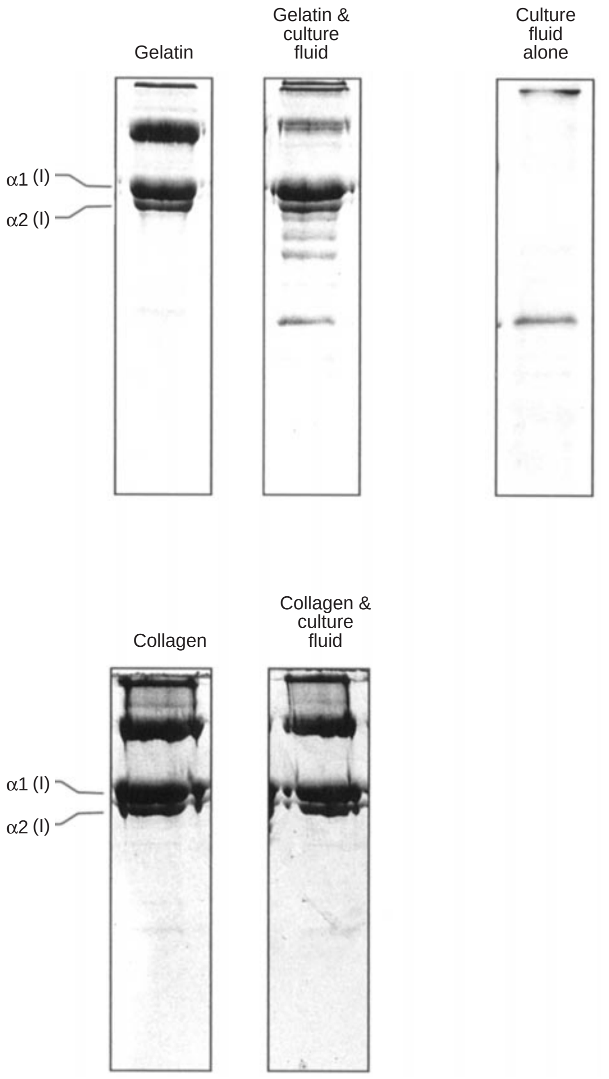

**Figure 4** Gelatinolytic and collagenolytic activity assessed by substrate-degradation assays in 72-hour prostate organ culture fluids. Intact gelatin and intact collagen are represented by the presence of intact α1(I) and α2(I) chains and the absence of lower molecular weight fragments. In the presence of 10×-concentrated and APMA-activated prostate organ culture fluid, there is degradation of gelatin as indicated by the presence of lower molecular weight bands. Such bands are not observed when collagen is exposed to the same culture fluid

### DISCUSSION

In the present study we have assessed the expression of MMPs with collagenolytic and gelatinolytic activity in fresh prostate tumour specimens and in organ cultures established from the same specimens. The results are discussed in relation to a number of issues including: i) the variability among different specimens, and the variability seen with different methods of assay; ii) alterations that occur in prostate tissue structure/function over time in organ culture, the overall levels of collagenolytic and gelatinolytic enzymes elaborated in organ culture, and the possible role of MMPs in these alterations; and iii) the implication of the findings for prostate tumour pathophysiology.

Considerable variability in MMP expression was observed among the 22 specimens analysed. In approximately half of the fresh tissues, MMP-2 and/or MMP-9 were not clearly identifyable by immunostaining. Except for an occasional reactive cell, MMP-1 was never routinely detected in this way, while MMP-13 was detected in the vasculature of virtually every specimen. In short-term (2–3 day) organ culture, MMP-1, as well as MMP-2 and MMP-9, was detected, but MMP-13 was not. Variability among specimens was due, in part we believe, to the heterogeneity in prostate tumour presentation, and to the amount of tumour present in any given tissue piece, as well as to intrinsic differences among tumours in enzyme elaboration. Some of the tumours demonstrated relatively well-differentiated features (Gleason scores of 4 or 5) while at the other extreme were tissues with highly anaplastic features. Equally important, the estimated percentage of the tissue occupied by tumour ranged from 1% to greater than 90%. Given this heterogeneity in the starting material, it is not surprising that there should be differences in MMP detection.

Although heterogeneity in prostate tumour presentation undoubtedly accounts for some of the variability noted, differences in analytical approaches also contribute to the variable results. It is clear from this work that even after incubation for as little as 2–3 days in organ culture, the culture medium from virtually every specimen contained detectable MMP-1, MMP-2 and MMP-9 as measured by zymography and Western blotting. These analytical techniques, particularly zymography are extremely sensitive (down to picogram quantities) (Gibbs et al, 1999) so it may be that enhanced enzyme detection reflects the increased sensitivity of the procedures used. Alternatively, differences in enzyme expression patterns between fresh tissue and organ-cultured tissue may reflect physiological differences. Consistent with our past report (Varani et al, 1999), normal basal epithelial cells underwent a proliferative response in organ culture under the conditions employed here while the majority of tumour cells lysed under the same conditions. The differences in enzyme recovery between fresh tissue specimens and short-term organ culture may, therefore, reflect a loss of malignant cells and a concomitant up-regulation of MMP production in association with the proliferative response in the normal component. The same signalling events that lead to epithelial cell proliferation also induce MMP production via AP-1 activation (Zeigler et al, 1999). Some of the variability in previously published reports (Lokeshwar et al, 1993; Stearns and Wang, 1993; Wilson et al, 1993; Hamdy et al, 1994; Montironi et al, 1995, 1996; Festuccia et al, 1996; Jung et al, 1997; Upadhyay et al, 1999; Still et al, 2000) may also have a similar basis. It should be noted that although we attribute the increase in MMP expression in organ culture primarily to the action of proliferating basal (non-malignant) epithelial cells, the presence of 'occult' tumour in the tissue cannot be completely ruled out. The presence of occult tumour would also be expected to contribute to the MMP levels observed in organ culture.

Two other issues need to be addressed. The first is the potential relationship between the MMPs elaborated by prostate tissue in organ culture and the changes in tissue structure/function which occur during organ culture. Although we were able to detect MMP-1 as well as MMP-2 and MMP-9 in organ culture fluid, and although the levels of MMP-1 and MMP-9 increased over time in culture, the actual amounts of all 3 enzymes in the tissue were, in fact, very low. This can be seen by the lack of active forms of the enzymes. Only faint, thin bands corresponding to active forms of both MMP-9 and MMP-2 were detected in gelatin zymograms while active forms of MMP-1 were completely undetectable in β-casein zymograms. Consistent with this, there was minimal

© 2001 Cancer Research Campaign 

gelatin degradation and no detectable collagen degradation in specific substrate assays.

The low level of enzyme activity in organ culture may be of interest in light of the changes that occurred in prostate tissue over time in culture. These included a proliferative response in the basal epithelial cell population and a concomitant loss of the malignant component (Schrodt and Foreman, 1971; Geller et al, 1992; Nevalainen et al, 1993; Pretlow et al, 1995; Varani et al, 1999; as well as the present report). The present study also demonstrated migration of the proliferating epithelial cells out from the gland across the surface of the tissue. This occurred wherever a cut in the tissue placed glandular epithelial cells in contact with the stroma. Basal cells, identified by their intense staining with anti-keratin (K903) antibody, could be seen covering the entire surface of the tissue pieces by day 8. Keratin-positive cells could also be seen wherever a break within the tissue was observed (Figure 2A, C). In spite of these changes, there was little evidence for stromal invasion. Basement membrane erosion (noted as a loss of staining with PAS or PAS-methenamine silver) was observed around some of the glands, but this was not a prominent feature in any of the tissues and (when assessed semi-quantitatively) not statistically different from what was observed in fresh tissue specimens. Additionally, detailed examination of sections from organ-cultured tissue failed to demonstrate areas in which actual epithelial cell penetration of the stroma had occurred.

In regard to these invasion-related events, the features observed in prostate organ culture are significantly different from what we have reported previously in organ cultures of normal human skin. Exposure of organ-cultured skin to the same peptide growth factors used here resulted in a dramatic increase in MMP production. In parallel, damage to the dermo-epidermal basement membrane occurred over a wide area, and epithelial cells penetrated deep into the stroma as columns of cells and as single cells. Basement membrane damage and stromal invasion were both inhibited by treatment with MMP inhibitors. Although these events occurred in the normal epidermal keratinocyte population, they provide a unique insight into the relationship between MMP up-regulation and stromal invasion under in situ conditions (Fligiel and Varani, 1993; Varani et al, 1995; Zeigler et al, 1996; Chi et al, 1998). In a subsequent study, it was shown that the phenotype expressed in normal skin following exposure to growth factors was indistinguishable from that expressed endogenously in basal cell carcinoma tissue (Varani et al, 2000). Based on comparison with these previous findings in skin, the results presented here allow us to speculate that the low level of MMP production in prostate tumour tissue contributes, at least in part, to the lack of tissue destruction and invasion in prostate organ culture. One might speculate further that the lack of a dense fibrous stroma in the prostate and the lack of significant collagenolytic enzyme activity are developmentally related.

In a similar manner, we can suggest that the relative lack of tissue destruction and stromal invasion in prostate carcinoma in situ may be attributable, at least in part, to the dearth of MMP activity in prostate tumour tissue. In other tumours, where these enzymes are elaborated in large amounts (for example, in basal cell tumours of skin), tissue destruction and stromal invasion are prominent features (Bauer et al, 1997; Goslen and Bauer, 1986). Thus, the fundamentally different behaviour of prostate tumours as compared to carcinomas of skin may be due, at least in part, to the low level of tissue-destructive enzymes in the prostate. This is not to imply, of course, that prostate carcinomas are completely non-invasive or destructive. As with all malignant tumours there is some level of tissue destruction and invasion. MMPs elaborated during the development of the disease (although in small amount as compared to some other tumours) may contribute to the disease process. Even without significant amounts of enzymes capable of degrading interstitial stroma, the gelatinases (MMP-2 and MMP-9) have the capacity to degrade basement membrane (type IV) collagen (Muschel et al, 1985), and elevated expression has been linked to malignant behaviour in other human tumours (Pyke et al, 1992).

In summary, a variety of approaches were used in this study to assess elaboration of gelatinolytic and collagenolytic MMPs by human prostate tumour tissue. There are 2 major findings from this work. First, results obtained with fresh tissue and with organ-cultured prostate are different. This can be attributed, at least in part, to the fact that the normal epithelial cell component proliferates in organ culture and is likely responsible for increased enzyme elaboration. Second, the overall levels of gelatinolytic and collagenolytic activities are low in prostate tumour tissue. This may explain, in part, the lack of tissue destruction and the failure of many prostate tumours to aggressively invade the surrounding stroma.

### ACKNOWLEDGEMENTS

This study was supported in part by grant CA60958 from the USPHS, by a Development Grant from the Prostate SPORE of the University of Michigan Comprehensive Cancer Center, and by a grant from the Veterans Education and Research Association of Michigan.

### REFERENCES

Alexander CM and Werb Z (1992) Targeted disruption of the tissue inhibitor of metalloproteinases gene increases the invasive behavior of primitive mesenchymal cells derived from embryonic stem cells in vitro. *J Cell Biol* **118**: 727–739

Azzam HS, Arand G, Lippman ME and Thompson EW (1993) Association of MMP-2 activation potential with metastatic progression in human breast cancer cell lines independent of MMP-2 production. *J Natl Cancer Inst* **85**: 1758–1764

Bauer EA, Gordon JM, Reddick ME and Eisen AZ (1977) Quantitation and immunochemical localization of human skin collagenase in basal cell carcinoma. *J Invest Dermatol* **69**: 363–367

Cha H-J, Bae S-K, Lee HY, Sato H, Seiki M, Park C and Kim K-W (1996) Anti-invasive activity of urosolic acid correlates with the reduced expression of matrix metalloproteinase-9 (MMP-9) in HT1080 human fibrosarcoma cells. *Cancer Res* **56**: 2281–2284

Chi Y, Zeigler ME, Walker J, Perone P, Datta SC and Varani J (1998) Elaboration of matrix metalloproteinase inhibitors by human skin in organ culture and by skin cells in monolayer culture: Relationship to invasion. *Invasion & Metastasis* **18**: 27–34,

Crawford HC and Matrisian LM (1996) Mechanisms controlling the transcription of matrix metalloproteinase genes in normal and neoplastic cells. *Enzyme Protein* **49**: 20–37

DeClerck YA, Yean T-D, Chan D, Shimada H and Langley KE (1991) Inhibition of tumor invasion of smooth muscle cell layers by recombinant human metalloproteinase inhibitor. *Cancer Res* **51**: 2151–2157

DeClerck YA, Perez N, Shimada H, Boone TC, Langley KE and Taylor SM (1992) Inhibition of invasion and metastasis in cells transfected with an inhibitor of metalloproteinases. *Cancer Res* **52**: 701–708

Festuccia C, Bologna M, Vicentini C, tacconelli A, Miano R, Violini S and Mackay AR (1996) Increased matrix metalloproteinase-9 secretion in short-term cultures of prostatic tumor cells. *Int J Cancer* **69**: 386–393

Fisher GJ, Datta SC, Talwar HS, Wang Z-Q, Varani J, Kang S and Voorhees JJ (1996) Molecular basis of sun-induced premature skin ageing and retinoid antagonism. *Nature (London)* **379**: 335–339

Fligiel SEG and Varani J (1993) In situ epithelial cell invasion in organ culture. *Invasion & Metastasis* **13**: 225–233

Geller J, Sionit LR, Connors K and Hoffman RM (1992) Measurement of androgen sensitivity in the human prostate in vitro three dimensional histoculture. *Prostate* **21**: 269–278

 *© 2001 Cancer Research Campaign*

Gibbs DF, Warner RO, Weiss SJ, Varani J and Johnson KJ (1999) Role of matrix metalloproteinases in lung injury in models of macrophage-dependent acute lung injury: Evidence for the macrophages as the source of the proteinases. *Amer J Respiratory Cell Molecular Biol* **20**: 1145–1154

Goslen JB and Bauer EA (1986) Basal cell carcinoma and collagenase. *J Dermatol Surg Oncol* **12**: 812–817

Gray ST, Wilkins RJ and Yun K (1992) Interstitial collagenase gene expression in oral squamous cell carcinoma. *Amer J Pathol* **141**: 301–306

Hamdy FC, Fadlon EJ, Cottain D, Lawry J, Thurrell W, Silcocks PB, Anderson JB, Williams JL and Rees RC (1994) Matrix metalloproteinase 9 expression in primary prostatic adenocarcinoma and benign prostatic hypeplasia. *Br J Cancer* **69**: 177–182

Imren S, Kohn DB, Shimad H, Blavier L and DeClerck YA (1966) Overexpression of tissue inhibitor of metalloproteinases-2 by retroviral-mediated gene transfer in vivo inhibits tumor growth and invasion. *Cancer Res* **56**: 2891–2895

Johansson N, Airola K, Grenman R, Kariniemi A-L, saarialho- Kere U and Kahari V-M (1997) Expression of collagenase-3 (MMP-13) in squamous cell carcinomas of the head and neck. *Amer J Pathol* **151**: 499–508

Jung K, Nowak L, Lein M, Priem F, Schnorr D and Loening SA (1997) Matrix metalloproteinases 1 and 3, tissue inhibitor of metalloproteinase-1 and the complex of metalloproteinase-1/tissue inhibitor in plasma of patients with prostate cancer. *Int J Cancer* **74**: 220–223

Key G, Becker MH, Baron B, Duchrow M, Schluter C, Flad HD and Gerdes J (1993) New Ki-67-equivalent murine monoclonal antibodies (MIB-1-3) generated against bacterially expressed parts of Ki-67 cDNA containing three 62 base pair repetitive elements. *Lab Invest* **68**: 629–636

Khokha R and Denhardt DT (1989) Matrix metalloproteinases and tissue inhibitors of metalloproteinases: a review of their role in tumorigenicity and tissue invasion. *Invasion & Metastasis* **9**: 391–405

Khokha R, Waterhouse P, Yagel S, Lala P, Overall C, Norton G and Denhardt D (1989) Antisense RNA-induced reduction in TIMP levels confers oncogenicity on Swiss 3T3 cells. *Science* **244**: 947–950

Lokeshwar BL, Selzer MG, Blook NL and Gunja-Smith Z (1993) Secretion of matrix metalloproteinases and their inhibitors (tissue inhibitor of metalloproteinases) by human prostate in explant culture: Reduced tissue inhibitor of metalloproteinase secretion by malignant tissues. *Cancer Res* **53**: 4493–4498

MacDougall JR, Bani MR, Lin Y, Rak J and Kerbel RS (1995) The 92 kDa gelatinase B is expressed by advanced stage melanoma cells: suppression by somatic hybridization with early stage melanoma cells. *Cancer Res* **55**: 4174–4181

Majmudar G, Nelson B, Jensen TC, Voorhees JJ and Johnson TM (1994) Increased expression of stromelysin-3 in basal cell carcinomas. *Mol Carcinogen* **9**: 17–23

Makin CA, Babrow LG and Bodner WF (1984) Monoclonal antibody to cytokaratin for use in routine histopathology. *J Clin Pathol* **37**: 395–404

McDonnell S, Navre M, Coffey RJ, jr. and Matrisian LM (1991) Expression and localization of the matrix metalloproteinase pump-1 in human gastric and colon carcinomas. *Mol Carcinogen* **4**: 527–533

Montironi R, Fabris G, Lucarini G and Biagini G (1995) Location of 72-kd metalloproteinase (type IV collagenase) in untreated prostatic adenocarcinoma. *Path Res Pract* **191**: 1140–1146

Montironi R, Lucarini G, Castaldini C, Galluzzi CM, Biagini G and Fabris G (1996) Immunohistochemical evaluation of type IV collagenase (72-kd metalloproteinase) in prostatic intraepithelial neoplasia. *Anticancer Res* **16**: 2057–2062

Muller D, Breathnach R, Englelmann A, Millon R, Bronner G, Flesch H, Dumont P, Eber M and Abecassis J (1991) Expression of collagenase-related metalloproteinase genes in human lung or head and neck tumours. *Int J Cancer* **48**: 550–556

Muller D, Wolf C, Abecassis J, Millon R, Engelmann A, Bronner G, Rouyer N, Rio M, Eber M, Methlin G, Chambon P and Basset P (1993) Increased stromelysin-3 gene expression is associated with increased local invasiveness in head and neck squamous cell carcinomas. *Cancer Res* **53**: 165–169

Mulligan MS, Desrochers PE, Chinnaiyan AM, Gibbs DF, Varani J, Johnson KJ and Weiss SJ (1993) In vivo suppression of immune complex-induced alveolitis by secretory leukoproteinase inhibitor and tissue inhibitor of metalloproteinase-2. *Proc Nat Acad Sci* (USA) **90**: 11523–11527

Murphy G and Docherty AJP (1992) The matrix metalloproteinases and their inhibitors. *Am J Respir Cell Mol Biol* **7**: 120–125

Murray GI, Duncan ME, O'Neil P, Melvin WT and Fothergill JE (1996) Matrix metalloproteinase-1 is associated with poor prognosis in colorectal cancer. *Nature Med* **2**: 461–462

Muschel R, Williams J, Lowy D and Liotta L (1985) Harvey ras induction of metastatic potential depends upon oncogene activation and the type of recipient cell. *Am J Pathol* **121**: 1–8

Nevalainen MT, Harkonen PL, Valve EM, Ping W, Nurmi M and Martikainen PM (1993) Hormone regulation of human prostate in organ culture. *Cancer Res* **53**: 5199–5207

Pajouh MS, Nagle RB, Breathnach R, Finch JS, Brawer MK and Bowden GT (1991) Expression of metalloproteinase genes in human prostate cancer. *J Cancer Res Clin Oncol* **117**: 144–150

Pretlow TG, Yang B and Pretlow TP (1995) Organ culture of benign, aging and hyperplastic human prostate. *Microscopic Res Tech* **30**: 271–281

Pyke C, Ralfkiaer E, Huhtala P, Hurskainen T, Dano K and Tryggvason K (1992) Localization of messenger RNA for $M_r$ 72,000 and 92,000 type IV collagenase in human skin cancers by in situ hybridization. *Cancer Res* **532**: 1336–1341

Schrodt GR and Foreman CD (1971) In vitro maintenance of human hyperplastic prostate tissue. *Invest Urol* **9**: 85–94

Stearns ME and Wang M (1993) Type IV collagenase (Mr 72,000) expression in human prostate: Benign and malignant tissue. *Cancer Res* **53**: 878–883

Still K, Robson CN, Autzen P, Robinson MC and Hamdy FC (2000) Localization and quantitation of mRNA for matrix metalloproteinase-2 (MMP-2) and tissue inhibitor of matrix metalloproteinase-2 (TIMP-2) in human benign and malignant prostatic tissue. *The Prostate* **42**: 18–25

Upadhyay J, Shekarriz B, Nemeth JA, Dong Z, Cummings GD, Fridman R, Sakr W, Grignon DJ and Cher ML (1999) Membrane type 1-matrix metalloproteinase (MT1-MMP) and MMP-2 immunolocalization in human prostate: Change in cellular localization associated with high-grade prostatic intraepithelial neoplasia. *Clin Cancer Res* **5**: 4105–4110

Varani J, Perone P, Inman DR, Burmeister W, Scholenberger SB, Fligiel SEG, Sitrin RG and Johnson KJ (1995) Human skin in organ culture: Elaboration of proteolytic enzymes in the presence and absence of exogenous growth factors. *Amer J Pathol* **146**: 210–217

Varani J, Dame MK, Wojno K, Schuger L and Johnson KJ (1999) Characteristics of nonmalignant and malignant human prostate in organ culture. *Lab Invest* **79**: 723–731

Varani J, Hattori Y, Chi Y, Schmidt T, Zeigler ME, Fader D and Johnson TM (2000) Collagenolytic and gelatinolytic matrix metalloproteinases and their inhibitors in basal cell carcinoma of skin: Comparison with normal skin. *Brit J Cancer* **82**: 657–665

Wilson MJ, Norris H, Kapoor D, Woodson M, Limas C and Sinha AA (1993) Gelatinolytic and caseinolytic proteinase activities in human prostate secretions. *J Urology* **149**: 653–658

Zeigler ME, Dutcheshen NT, Gibbs DFG and Varani J (1996) Growth factor-induced epidermal invasion of the dermis in human skin organ culture: Expression and role of matrix metalloproteinases. *Invasion & Metastasis* **16**: 11–18

Zeigler ME, Chi Y, Schmidt T and Varani J (1999) Role of ERK and JNK pathways in regulating cell motility and matrix metalloproteinase 9 production in growth factor-stimulated human epidermal keratinocytes. *J Cell Physiol* **180**: 271–284

© 2001 Cancer Research Campaign
